# Comparative study of microbial structure and functional profile of sunflower rhizosphere grown in two fields

**DOI:** 10.1186/s12866-021-02397-7

**Published:** 2021-12-09

**Authors:** Blessing Chidinma Nwachukwu, Ayansina Segun Ayangbenro, Olubukola Oluranti Babalola

**Affiliations:** Food Security and Safety Niche, Faculty of Natural and Agricultural Science, Private Mail Bag X2046, North-West University, Mmabatho, 2735 South Africa

**Keywords:** Amplicon sequencing, Biotechnology, Community-level physiological profiling (CLPP), Rhizosphere microbiome, Sustainable agriculture

## Abstract

**Background:**

Microbial communities inhabiting the rhizosphere play pivotal roles in determining plant health and yield. Manipulation of the rhizosphere microbial community is a promising means to enhance the productivity of economically viable and important agricultural crops such as sunflower (*Helianthus annuus*). This study was designed to gain insights into the taxonomic and functional structures of sunflower rhizosphere and bulk soil microbiome at two different locations (Sheila and Itsoseng) in South Africa.

**Results:**

Microbial DNA extracted from the sunflower rhizosphere and bulk soils was subjected to next-generation sequencing using 16S amplicon sequencing technique. *Firmicutes*, *Actnobacteria* and *Proteobacteria* predominated sunflower rhizosphere soils. *Firmicutes*, *Cyanobacteria*, *Deinococcus*-*Thermus* and *Fibrobacteres* were positively influenced by Na^+^ and clay content, while *Actinobacteria*, *Thaumarchaeota*, *Bacteroidetes*, *Planctomycetes*, *Aquificae* and *Chloroflexi* were positively influenced by soil resistivity (Res) and Mg^2+^. The community-level physiological profiling (CLPP) analysis showed that the microbial communities in SHR and ITR used the amino acids tryptophan and malic acid efficiently. The metabolisms of these carbon substrates may be due to the dominant nature of some of the organisms, such as *Actinobacteria* in the soils.

**Conclusion:**

The CLPP measurements of soil from sunflower rhizosphere were different from those of the bulk soil and the degree of the variations were based on the type of carbon substrates and the soil microbial composition. This study has shown the presence of certain taxa of rhizobacteria in sunflower rhizosphere which were positively influenced by Na^+^ and Mg^2+^, and taxa obtained from SHR and ITR were able to effectively utilized tryptophan and malic acid. Many unclassified microbial groups were also discovered and it is therefore recommended that efforts should further be made to isolate, characterize and identify these unclassified microbial species, as it might be plausible to discover new microbial candidates that can further be harnessed for biotechnological purpose.

## Background

Microbial communities inhabiting the rhizosphere play pivotal roles in plant health and are responsible for sustaining soil health and functions [1, 2]. These microbes provide essential nutrients, such as N and P, required for plant use, and up to 40–50% of N and 75% of P required by plants are supplied yearly [3]. Hence, the manipulation of the rhizosphere microbial community is a promising means to improve the productivity of agricultural crops [4].

In previous studies, soil type and introduction of alien microbes have been shown to contribute to the distribution of rhizosphere microbial communities [5, 6]. Other factors that are drivers of microbial community composition are plant species, plant developmental stages, climatic conditions, and the interplay between these factors [7, 8]. For example, studies on the rhizosphere of *Baphicacanthus cusia* [9] and *Jacobaea vulgaris* [10] showed that the rhizosphere zones are hotspots for microbial growth, abundance and diversity as they offer habitats with increased nutrient availability. The studies further showed that though plants have a strong selection impact on the rhizosphere microbiome, soil type has an effect on their stability and composition [11]. observed that the host genotype of grapevine rootstock genotypes did not predict any precise metrics of rhizosphere alpha and beta biodiversity in the young vineyard, whilst it was reported that various rhizosphere microbiome are associated with grapevine rootstock genotypes in the matured vineyard. Consequently, the latter could have been directly influenced by soil properties, age of the vineyard or agricultural management practices, which are key drivers of the rhizosphere microbiome in agricultural ecosystems.

Furthermore, plant roots select microbes inhabiting the rhizosphere region by secreting different metabolites, which vary with plant age and species, including secondary metabolites such as phytohormones and antimicrobial compounds that induce defense against phytopathogens [12]. To also select microbial species, plant roots create a discrete microenvironment by adjusting the oxygen and pH concentrations in the rhizosphere [13]. In recent times our knowledge of the rhizosphere microbiome has advanced. Many aspects of these rhizosphere microbial communities assemblages are less understood, especially in plants cultivated on the field [14].

In addition, limited studies have been conducted on the rhizosphere of valuable agronomic crops such as sunflower plants (*Helianthus annuus*). Sunflower is essential for oil production and has many benefits to humans [4, 15]. It is, therefore, necessary to investigate the rhizosphere of sunflower plants in the field with the aim of determining microbial species with beneficial plant growth-promoting traits that can be used to improve the growth and health of sunflower plants. Thus, in this study, we evaluated the functional and microbial diversity of sunflower rhizosphere grown in the field in two different locations (Sheila and Itsoseng) in Northwest province, South Africa.

## Materials and methods

### Study sites and sample collection

**Fig. 1:**
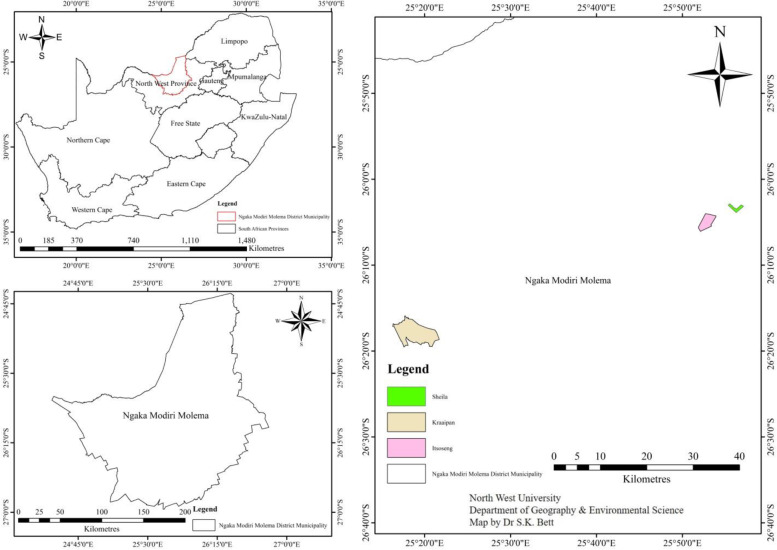
Map of the study area in South Africa showing Ngaka Modiri Molema district municipality (bottom left) where the two farms Sheila (green) and Itsoseng (pink) are located

The cultivar Pen 7011 Pannar was planted in the two sunflower fields. The study sites were located at Sheila (SH) (26°2′41.202″ S, 25°57′47.49″ E) and Itsoseng (IT) (26°4′23.064″ S, 25°58′37.104″ E) situated at Ngaka Modiri Molema district municipality, North-West Province, South Africa (Fig. 1). North-West Province has a mean annual rainfall of 300–700 mm annually. The sunflower farm at Sheila has been planted solely with maize for the past 5 years, a farming system called monoculture system, however, the Itsoseng farm has been subjected to crop rotation system for the past 5 years. In particular, maize-sunflower-pea-oat-sunflower have been rotationally planted on the Itsoseng farm. In addition, both sunflower farms have a history of maintaining conventional agronomic management practices, including pest control, weed control and NPK (15:8:4) fertilizer application.

We received the approval of the owners/farmers before the sunflower rhizosphere and bulk soil samples were collected from the farms. Sunflower rhizosphere soil samples were collected randomly from plants at different points on the farms by uprooting the sunflower plants. The roots were shaken to remove the loosely attached soil particles. The bulk soil samples, which serve as the control, were collected 10 m away at a depth of about 0–20 cm from the plant root using a soil auger. Two sites were selected for each of the two farms. At each of the two sites, three rhizosphere and three bulk soil samples were aseptically collected and pooled into labelled sterile plastic bags and were homogenized to obtain a single composite sample as described by [2]. Soil samples for the molecular analysis were stored − 80 °C before processing.

### Determination of soil physical and chemical properties

Rhizosphere and bulk soil samples analyses were performed at Agricultural Research Council-Soil, Climate and Water laboratory, Arcadia, Hatfield Pretoria, South Africa, using standard procedures.

Before soil analyses, soil samples were air-dried and sieved through a 2 mm mesh to remove roots debris and large rocks and dried overnight at 50 °C. The pH (1:3 soil/deionized water) of the soil samples were evaluated by a slurry technique using a Crison Bench pH meter (Crison Instruments, Barcelona, Spain) after allowing the soil to settle for 30 min. The soil particle size was classified through the hydrometer technique [16]. The total nitrogen and carbon were analyzed using the dry combustion method previously described by [17]. Phosphorus was determined with a spectrophotometer [18]. Organic matter (OM) in the soil was measured by Walkley Black method [19]. Soil nitrate was measured by KCI extraction method. Soil calcium, potassium, magnesium, and sodium were evaluated using 1 M ammonium acetate at pH 7.0 [20]. Subsequently, magnesium, calcium, and sodium in the extracts were determined using atomic absorption spectrophotometer, while a flame photometer was used to measure the potassium [20, 21].

### DNA extraction and 16S rDNA amplicon sequencing

After removing the pebbles by sieving through 2 mm sieve, the microbial DNA was extracted from 0.5 g each of the soil samples collected from the sunflower rhizosphere and bulk soils using the Quick-DNA isolation kit (Zymo Research, Irvine, USA) following the manufacturer’s instruction. DNA samples were sequenced at the Molecular Research Laboratory (Texas, USA).

Briefly, polymerase chain reactions (PCRs) were conducted in a single-step PCR using HotStarTaq Plus Master Mix Kit (Qiagen, USA) with primer pairs 515F (5′- AATGATACGGCGACCACCACCGAGATCTACAC TATGGTAATT GT GTGCCAGCMGCCGCGGTAA-3′) and 806R (5′-CAAGCAGAAGACGGCATACGAGAT TCCCTTGTCTCCAGTCAGTCAG CC GGACTACHVGGGTWTCTAAT-3′), which amplified both archaea and bacteria. The PCR products from all DNA samples were quantified using PicoGreen dsDNA assay. The samples were pooled together in equimolar concentration and then, purified using calibrated Ampure XP beads (Agencourt Bioscience Corporation, MA, USA). The pooled and the purified PCR product was used to prepare an Illumina DNA library. Sequencing was performed on an Illumina MiSeq 2000 using a paired-end technique to obtain 312 bp paired-ends reads.

### Functional measurements using community-level physiological profiling (CLPP) technique

MicroResp™, as described by [22], was used to determine community respiration and substrate-induced respiration. Approximately 0.4 g of soil samples were placed in each well of 96-deep well plates, and incubated for 2 days at room temperature in the dark before the assay was performed. A total of 11 carbon substrates, including three amino acids (methionine, tyrosine, and tryptophan), five carbohydrates (galactose, maltose, glucose, fructose, and sucrose), and three carboxylic acids (D-pantothenic acid, citric acid, and malic acid) were selected. The choice of substrates was based on substrates complexity (i.e., carbon chain length) [23, 24]. All the carbon substrates used in this study were purchased from Sigma-Aldrich (Australia). Substrates were dissolved in deionized water, sterilized by filtering and introduced to four replicated wells. Preceding the addition of substrates, CO_2_ detection microplates containing 12.5 μg mL-^1^ cresol red, 2.5 mM sodium bicarbonate and 150 mM potassium chloride were pre-read at absorbance wavelength of 570 nm, then the deep-well plates were immediately placed on the airtight MicroResp™ seal and incubated at 25 °C for 6 h in the dark in a desiccator, as recommended by the manufacturer (Macaulay Scientific Consulting, UK). The differences in optical density after incubation was then estimated on a spectrophotometer microplate reader (EnSpire® 2300 Multilabel Reader, Perkin Elmer, USA) at a wavelength of 570 nm. The quantity of CO_2_ respiration per gram of soil per well was calculated by adopting the formula in the MicroResp™ manual (James Hutton Ltd., UK), the rate of CO_2_ respiration expressed per gram of soil was estimated.

### Data processing and/or bioinformatics analysis

The raw sequenced data were processed using MR DNA analysis pipeline (MR DNA, Shallowater, TX, USA). In summary, paired ends of DNA sequences were combined, ‘depleted of barcode’ and sequences less than 150 base pairs and vague base call were jettisoned. DNA sequences were later denoised, operational taxonomic units (OTUs) were produced and chimeras were removed. OTUs were classified by grouping at 3% divergence (97% similarity) and defined into a taxonomy using ‘BLASTn against curated database’ obtained from RDPII and NCBI (http: //rdp.cme.msu. Edu, https://www.ncbi.nlm.nih.gov).

Processed sequenced data were subsequently uploaded to MG-RAST for analyses [25]. The analyzed data were downloaded from MG-RAST and pasted on a Microsoft Excel sheet. The species richness was estimated using rarefaction obtained from the MG-RAST pipeline. Alpha diversity was expressed using Shannon and evenness indices, while the Kruskal-Wallis test was used to describe the diversity indices across sites, and all the analyses were performed using PAST version 3.20 [26]. Taxonomic richness was represented as an OTU number. The heatmap expressing the relative abundance of microbial communities at the phylum level was plotted using Shinyheatmap [27]. The principal coordinates analysis (PCoA) was used to analyze the abundance data of relative OTU i.e. the microbial structure based on Bray-Curtis distances using CANOCO 5 (Microcomputer Power, Ithaca, NY, USA). The correlation between the measured physical/chemical properties and microbial communities was estimated using the canonical correspondence analysis (CCA) method on CANOCO 5 software. The forward selection of environmental factors and the Monte Carlo permutation test were used to evaluate the environmental variables that best described the microbial composition. The 999 random permutations were employed for the significance test. The environmental variables itemized in Table 1 were used as explanatory variables in the CCA analysis.

**Table 1:** Physical and chemical properties of the sunflower soils

Sample	SHR	SHB	ITR	ITB
OM (%)	1.98 ± 0.0^bc^	2.11 ± 0.1^b^	1.78 ± 0.1^c^	2.81 ± 0.0^a^
N-NO_3_ (mg/kg)	12.31 ± 2.8^ab^	13.27 ± 0.2^a^	5.54 ± 2.5^bc^	4.30 ± 0.0 ^c^
N-NH_4_ (mg/kg)	4.93 ± 0.2^c^	4.59 ± 0.1^c^	6.67 ± 0.1^b^	11.79 ± 0.1^a^
pH	6.61 ± 0.1^a^	6.69 ± 0.0 ^a^	6.02 ± 0.1^b^	6.05 ± 0.0 ^b^
Res.(ohm)	1270 ± 3.0^b^	1230 ± 30.0^b^	2275 ± 155.0^a^	2020 ± 20.0^a^
P^3−^ (mg/kg)	68.50 ± 8.8^a^	81.29 ± 2.6^a^	14.09 ± 1.2^b^	79.84 ± 3^.^0^a^
Ca^2+^ (mg/kg)	560.50 ± 81.0^ab^	659.50 ± 14.5^a^	451.50 ± 3.5^b^	651.00 ± 17.0^a^
Mg^2+^ (mg/kg)	141.00 ± 3.0^a^	138.50 ± 3.5^a^	152.00 ± 8.0^a^	135.50 ± 3.5^a^
K^+^ (mg/kg)	161.50 ± 0.5^a^	169.00 ± 3.0^a^	119.00 ± 7.0^b^	167.00 ± 1.0^a^
Na^+^ (mg/kg)	78.70 ± 1.9^a^	71.40 ± 3.4^a^	71.95 ± 2.4^a^	70.35 ± 3.4^a^
C (%)	0.46 ± 0.0^b^	0.56 ± 0.1^b^	0.46 ± 0.0^b^	1.06 ± 0.1^a^
N^3−^ (%)	0.05 ± 0.0^b^	0.05 ± 0.0^b^	0.05 ± 0.0^b^	0.09 ± 0.0^a^
Sand (%)	78.00 ± 0.0^b^	78.00 ± 0.0^b^	79.00 ± 1.0^b^	84.00 ± 0.0^a^
Silt (%)	2.00 ± 0.0 ^a^	3.00 ± 1.0 ^a^	2.00 ± 0.0 ^a^	3.00 ± 1.0 ^a^
Clay (%)	20.00 ± 0.0^a^	19.00 ± 1.0^a^	19.00 ± 1.0^a^	13.00 ± 1.0^b^

Legend: *SHR* Sheila rhizosphere soil; *SHB* Sheila bulk soil; *ITR* Itsoseng rhizosphere soil; ITB- Itsoseng bulk soil. Number of replicates (n) = 3. Data represent mean ± SE. Mean values having the same alphabets are considered not statistically significant (P ≥ 0.05) following Duncan’s multiple range test

All the sequences for this study were deposited in the Sequence Read Archive (SRA) of the National Center for Biotechnology Information (NCBI) under the Bioproject number PRJNA672856.

### Statistical analysis

The mean and standard errors of the physical and chemical properties and the functional measurement data were obtained in a Microsoft Excel sheet. Physical and chemical properties data were transferred to SPSS, where one-way ANOVA and Duncan Multiple tests were carried out. Graphs and one-way ANOVA for functional measurements were performed using GraphPad Prism 7, GraphPad Software, California. For the functional measurements, two composite samples were used per farm and each composite sample was replicated four times in the wells of MicroResp™ plates upon addition of the different carbon sources. The mean of the four replicates per composite sample was obtained, and thereafter, the obtained mean values for the two composite samples per site were used for statistical analysis. Probability values less than or equal to 0.05 (*p* ≤ 0.05) was considered to be statistically significant for all the data.

## Results

### Soil physicochemical properties of the sunflower soil samples

The soil’s physical and chemical properties are presented in Table 1. The amount of N-NH_4_ in soil samples from SHR and SHB was not significantly different (*p* > 0.05), but N-NH_4_ content in soil from ITB was significantly different from soil from ITR (*p* ≤ 0.05). Similarly, rhizosphere soil did not influence soil pH in soil obtained from sunflower cultivated in Sheila since a significant difference was not observed in SHR and SHB. Also, ITR and ITB soil pH was not significantly different (*p* > 0.05). In addition, Mg^2+^, Na^+^ and silt (%) were not significantly different (*p* > 0.05) in all the soil samples from the two sites, while the ITR rhizosphere soil significantly influenced percentage clay (*p* ≤ 0.05) compared to ITR bulk soil.

### Rarefaction analysis

**Fig. 2:**
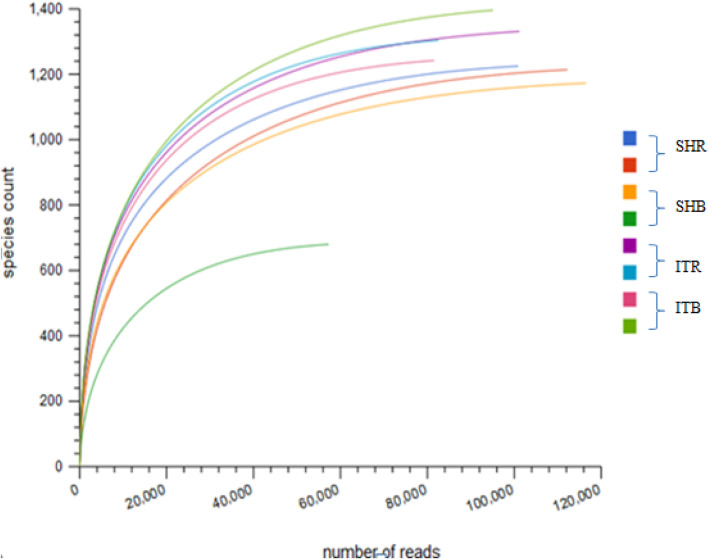
Rarefaction curves show the estimated richness in the rhizosphere soils and sampling effort. The total number of OTUs measured after sampling is represented on the y-axis, while the number of sequences is represented on the x-axis. Legend: SHR- Sheila rhizosphere soil, SHB- Sheila bulk soil, ITR- Itsoseng rhizosphere soil, ITB- Itsoseng bulk soil

The richness of the microbial diversity in the sunflower soil samples was measured by rarefaction analysis (Fig. 2). From the results, the rarefaction curves for Sheila soils (SHR and SHB) were higher than the soils from Itsoseng (ITR and ITB). Most of the sample reads got to the saturation level, which indicates that the sampling methods were sufficiently covered (Fig. 2). From the rarefaction curve, the highest read number 120000 (x-axis) aligned with SHB, while 1200–1400 (y-axis) were the highest species counts. It was possible to subsample 120,000 reads from the SHB sample when according to Table 2 only 10,364 sequences were obtained for SHB because Table 2 reports the means of two samples for SHB.

**Table 2:** Analysis of sequenced data of the amplicon metagenomes of the sunflower soil samples

Sampling site	SHR	SHB	ITR	ITB
**Uploading Information**				
bp count	36,938,758	25,321,399	27,104,520	26,066,428
Sequences count	125,264.5	86,929.5	91,939.5	88,384.5
Mean sequence length (bp)	295 ± 5.0	289.5 ± 29.0	295 ± 5.0	295 ± 5.0
Mean GC count (%)	55 ± 3.0	53.5 ± 3.0	56.5 ± 3.0	57 ± 3.0
**Post QC Information**				
bp count	4,181,225	2,912,336.5	4,260,016	4,645,809
Sequences count	14,556	10,364	14,637.5	15,941
Mean sequence length (bp)	287.5 ± 24.0	279 ± 40.0	291 ± 9.0	291 ± 10.0
Mean GC count (%)	56 ± 4.0	54.5 ± 4.0	56.5 ± 4.0	57 ± 4.0
**Processed Sequences**				
Predicted proteins features	155.5	193.0	254.5	344.0
Predicted rRNA features	22,730	13,358.5	22,051	25,648
**Aligned Sequences**				
Identified protein features	60	60.5	98.5	125.5
Identified rRNA features	19,312.5	11,693.5	18,265	20,065

Legend: *SHR* Sheila rhizosphere soil, *SHB* Sheila bulk soil, *ITR* Itsoseng rhizosphere soil, *ITB* Itsoseng bulk soil, *GC* Guanine cytosine content, bp-base pair,, data represent the mean of the original samples

### Assessment of diversity indices

The RDP classifier was used to assign sequence reads of Sheila and Itsoseng soils into OTUs with 3% nucleotide cut-off values. The estimated Simpson, Shannon, evenness, and Chao-1 indices are presented in Table 2.

The pre-processed analytical output unveiled different parameters like bp count, sequences count, mean sequence length (bp) and mean GC count (%). The post-process information similarly entails bp count, sequences count, mean sequence length (bp) and mean GC count (%), including predicted protein and rRNA features (Table 2). The pre-processed bp count were 36,938,758 (SHR), 25,321,399 (SHB), 27,104,520 (ITR), and 26,066,428 (ITB), with mean sequence bp length of 295 ± 5.0 (SHR), 289.5 ± 29.0 (SHB), 295 ± 5.0 (ITR), and 295 ± 5.0 (ITB). Also, the sequence count of the pre-processed data comprises a mean sum of 125,264.5, 86,929.5, 91,939.5, and 88,384.5 for SHR, SHB, ITR and ITB, respectively, with a corresponding mean GC (%) count of 55 ± 3.0, 53.5 ± 3.0, 56.5 ± 3.0 and 57 ± 3.0 (Table 2).

Upon processing, a reduction in the bp count of the sequence reads was observed in all the soil samples with mean values corresponding to 4,181,225 (SHR), 2,912,336.5 (SHB), 4,260,016 (ITR), and 4,645,809 (ITB). A similar decrease was observed for the sequence count and mean sequence bp length in all the soil samples. However, 56 ± 4.0, 54.5 ± 4.0, 56.5 ± 4.0, and 57 ± 4.0 were the % mean GC count of post-processed or quality control data for the SHR, SHB, ITR and ITB, respectively (Table 2). Out of the sequence reads that passed pre-processing test, 60, 60.5, 98.5, and 125.5 sequence reads corresponding to SHR, SHB, ITR and ITB have predicted protein features with known functions, while others have protein features with unknown functions. Also, 19,312.5, 11,693.5, 18,265, and 20,065 sequence reads from SHR, SHB, ITR and ITB were found to have predicted protein features that code for known rRNA (Table 2).

### Alpha diversity assessment of the composition and abundance of taxonomic groups in sunflower rhizosphere and bulk soils

**Fig. 3:**
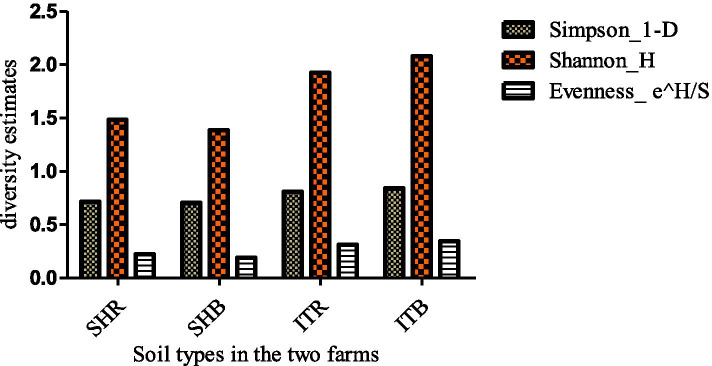
Diversity indicators of the abundance of the microbial taxonomic groups in sunflower rhizosphere and bulk soils. SHR- Sheila rhizosphere soil, SHB- Sheila bulk soil, ITR- Itsoseng rhizosphere soil, ITB- Itsoseng bulk soil

Statistical indicators such as Simpson, Shannon and evenness were used to describe alpha diversity (i.e., diversity within sunflower soil type) of the abundance of the microbial taxonomic groups in the different soil samples obtained from the different sunflower soil types. The Simpson, Shannon, and evenness indicators reveal that the microbial taxonomic groups show no significant differences (Kruskal–Wallis, *p*-value = 0.77) as represented in Fig. 3.

### Beta diversity assessment of the composition and abundance of taxonomic groups in sunflower rhizosphere and bulk soils

**Fig. 4:**
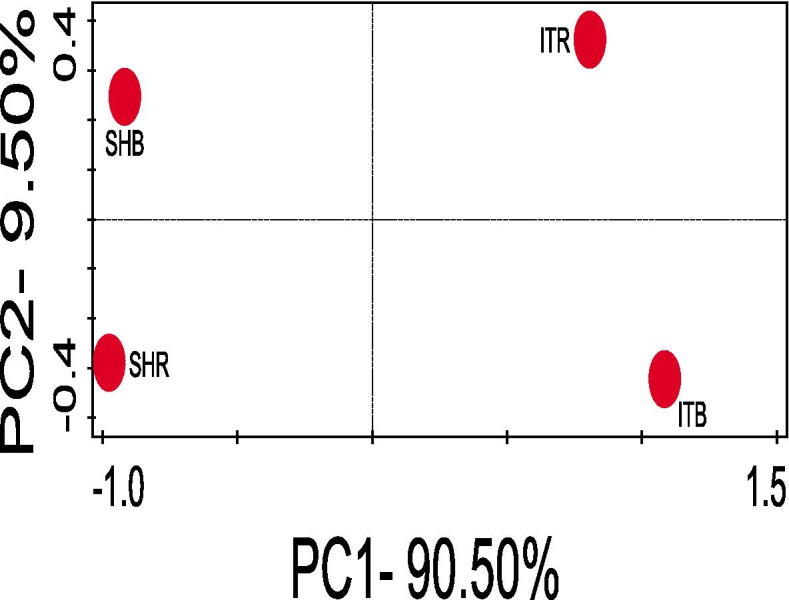
Principal coordinates analysis (PCoA) of the microbial (bacterial and archaeal) community composition and abundance at the operational taxonomic unit (OTU) level to illustrate beta diversity between sunflower rhizosphere soils and bulk soils obtained from Sheila and Itsoseng. SHR- Sheila rhizosphere soil, SHB- Sheila bulk soil, ITR- Itsoseng rhizosphere soil, ITB- Itsoseng bulk soil

On the other hand, a significant difference (ANOSIM, *p*-value = 0.01, R = 0.60) was observed in the beta diversity (i.e., diversity between the sunflower rhizosphere soil and bulk soil samples) of the soil microbial composition and abundance obtained from Sheila and Itsoseng as illustrated by Fig. 4 using principal coordinates analysis (PCoA).

### Microbial structures at the phylum and genus level observed in Sheila and Itsoseng rhizosphere and bulk soils

Sequence tags were assigned to Sheila, and Itsoseng rhizosphere and bulk soil samples into different taxa via rapid metagenomic annotations using subsystems technology (MG-RAST) at The relative abundance of microbial phyla for each soil sample is presented in Table 3. *Firmicutes* and *Proteobacteria* were observed to be abundant in both SHR and SHB, whereas *Actinobacteria* and *Proteobacteria* dominated ITR, while *Thaumarchaeota* and *Actinobacteria* predominated in ITB. The mean relative abundant values of 39.74 and 31.82 were observed for *Firmicutes* in SHR and SHB, respectively, while 29.47 and 33.68 were recorded for *Proteobacteria* in SHR and SHB, respectively. Also, *Actinobacteria* had a mean relative abundant value of 26.41 for ITR, which was higher than that of ITB (19.50), while *Thaumarchaeota* in ITB had a mean relative abundant value of 19.40. However, the mean relative abundant values of the entire microbial community in Table 3 were not significantly different (*p* > 0.05).

**Table 3:** The microbial taxonomic structure and mean relative abundance at phylum level from sunflower rhizosphere and bulk soils obtained from Sheila and Itsoseng farms

Phylum		Farm		
	SHR	SHB	ITR	ITB
*Firmicutes*	39.74 ± 6.54	31.82 ± 9.93	12.11 ± 8.56	9.54 ± 0.08
*Proteobacteria*	29.47 ± 0.55	33.68 ± 18.81	12.67 ± 8.96	11.49 ± 1.19
unclassified (derived from Bacteria)	19.29 ± 2.36	27.72 ± 13.15	28.97 ± 20.48	23.48 ± 1.97
*Actinobacteria*	6.22 ± 1.59	3.59 ± 2.71	26.41 ± 18.66	19.50 ± 1.29
*Bacteroidetes*	1.58 ± 0.91	0.83 ± 0.29	3.14 ± 2.22	3.75 ± 0.29
*Thaumarchaeota*	0.92 ± 0.08	0.49 ± 0.44	8.25 ± 5.83	19.40 ± 2.82
*Gemmatimonadetes*	0.51 ± 0.08	0.46 ± 0.21	0.83 ± 0.59	1.43 ± 0.17
unclassified (derived from unclassified sequences)	0.36 ± 0.24	0.23 ± 0.17	1.35 ± 0.95	1.16 ± 0.06
*Cyanobacteria*	0.26 ± 0.24	0.03 ± 0.01	0.07 ± 0.05	0.06 ± 0.00
*Acidobacteria*	0.37 ± 0.08	0.29 ± 0.07	1.68 ± 1.18	3.59 ± 0.01
*Verrucomicrobia*	0.31 ± 0.13	0.33 ± 0.07	2.23 ± 1.58	3.99 ± 0.61
*Planctomycetes*	0.22 ± 0.12	0.10 ± 0.03	0.34 ± 0.24	0.49 ± 0.02
*Chloroflexi*	0.22 ± 0.09	0.14 ± 0.11	0.66 ± 0.46	0.53 ± 0.10
*Nitrospirae*	0.16 ± 0.07	0.11 ± 0.08	0.36 ± 0.25	0.77 ± 0.00
*Crenarchaeota*	0.23 ± 0.03	0.07 ± 0.06	0.66 ± 0.47	0.11 ± 0.05
*Spirochaetes*	0.06 ± 0.00	0.04 ± 0.03	0.20 ± 0.14	0.53 ± 0.12
*Chlamydiae*	0.02 ± 0.02	0.01 ± 0.00	0.01 ± 0.01	0.05 ± 0.01
*Deinococcus*-*Thermus*	0.03 ± 0.01	0.03 ± 0.02	0.02 ± 0.01	0.01 ± 0.00
*Fibrobacteres*	0.02 ± 0.01	0.01 ± 0.01	0.00 ± 0.00	0.00 ± 0.00
*Tenericutes*	0.01 ± 0.00	0.01 ± 0.01	0.01 ± 0.01	0.01 ± 0.00
*Aquificae*	0.00 ± 0.00	0.00 ± 0.00	0.01 ± 0.01	0.01 ± 0.00
*Chlorobi*	0.00 ± 0.00	0.01 ± 0.01	0.00 ± 0.00	0.02 ± 0.00
*Dictyoglomi*	0.00 ± 0.00	0.00 ± 0.00	0.01 ± 0.01	0.07 ± 0.01
*Synergistetes*	0.00 ± 0.00	0.00 ± 0.00	0.01 ± 0.00	0.01 ± 0.01

Legend: *SHR* Sheila rhizosphere soil, *SHB* Sheila bulk soil, *ITR* Itsoseng rhizosphere soil, *ITB* Itsoseng bulk soil, data represent mean ± standard error of the relative abundance of microbial phyla

Furthermore, at the genus level, the microbes that were observed in the order of decreasing relative abundance include: *Bacillus*, *Pseudomonas*, *Staphylococcus*, *Vagococcus*, *Rubrobacter*, *Brevundimonas*, *Candidatus Nitrososphaera*, *Lysinibacillus*, *Terrimonas*, *Gemmatimonas*, *Paenibacillus*, *Mitsuaria*, *Clostridium*, *Streptomyces*, *Chroococcidiopsis*, *Sphingobacterium*, *Geodermatophilus*, *Alcaligenes*, *Oxalicibacterium*, *Candidatus Amoebophilus*, *Sphingomonas*, *Bradyrhizobium*, *Acinetobacter*, *Nocardioides*, *Cystobacter* and *Cellulomonas* (Table 4). A mean relative abundance of 34.65 for *Bacillus* was recorded in SHR as against 29.56 observed for *Pseudomonas* in SHB. Aligned to the microbial community structures at the phylum level, the mean relative abundant values of microbial species at the generic level did not show significance difference (*p* > 0.05).

**Table 4:** The microbial taxonomic structure and relative abundance at genus and/or generic level from sunflower rhizosphere and bulk soils obtained from Sheila and Itsoseng farms

Genus		Farm		
	SHR	SHB	ITR	ITB
*Bacillus*	34.65 ± 9.95	29.50 ± 10.39	9.91 ± 2.69	6.95 ± 0.68
unclassified (derived from Bacteria)	21.29 ± 3.08	29.16 ± 13.21	37.32 ± 10.83	33.79 ± 2.72
*Pseudomonas*	25.05 ± 1.71	29.56 ± 18.98	5.59 ± 1.83	1.67 ± 0.47
*Staphylococcus*	3.51 ± 3.28	0.21 ± 0.03	0.05 ± 0.02	0.01 ± 0.01
*Vagococcus*	2.02 ± 0.14	1.39 ± 0.28	0.53 ± 0.33	0.09 ± 0.01
*Arthrobacter*	1.56 ± 0.33	1.19 ± 1.03	8.30 ± 3.00	1.64 ± 0.03
*Rubrobacter*	1.07 ± 0.36	0.77 ± 0.59	10.74 ± 8.13	7.46 ± 1.74
*Brevundimonas*	1.21 ± 0.01	1.47 ± 0.39	0.50 ± 0.34	0.08 ± 0.02
*Candidatus Nitrososphaera*	1.02 ± 0.11	0.54 ± 0.48	10.48 ± 0.94	27.16 ± 2.22
unclassified (derived from *Betaproteobacteria*)	0.76 ± 0.29	1.12 ± 0.07	1.34 ± 0.79	1.56 ± 0.25
unclassified (derived from *Gammaproteobacteria*)	0.58 ± 0.26	0.47 ± 0.16	0.82 ± 0.19	1.13 ± 0.21
unclassified (derived from *Deltaproteobacteria*)	0.54 ± 0.27	0.49 ± 0.03	1.72 ± 0.29	2.71 ± 0.31
unclassified (derived from *Alphaproteobacteria*)	0.55 ± 0.19	0.53 ± 0.28	1.72 ± 0.19	2.48 ± 0.01
Lysinibacillus	0.48 ± 0.22	0.47 ± 0.04	0.18 ± 0.09	0.37 ± 0.08
*Terrimonas*	0.54 ± 0.14	0.30 ± 0.20	2.24 ± 0.62	2.95 ± 0.10
*Gemmatimonas*	0.57 ± 0.11	0.49 ± 0.24	1.06 ± 0.16	2.01 ± 0.21
unclassified (derived from unclassified sequences)	0.40 ± 0.27	0.25 ± 0.19	1.68 ± 0.25	1.64 ± 0.09
*Paenibacillus*	0.49 ± 0.10	0.33 ± 0.28	0.69 ± 0.29	0.16 ± 0.00
*Mitsuaria*	0.27 ± 0.27	0.00 ± 0.00	0.02 ± 0.01	0.04 ± 0.04
*Clostridium*	0.35 ± 0.15	0.16 ± 0.09	0.19 ± 0.02	0.57 ± 0.20
*Streptomyces*	0.32 ± 0.12	0.19 ± 0.16	1.12 ± 0.35	2.79 ± 0.47
*Chroococcidiopsis*	0.21 ± 0.21	0.00 ± 0.00	0.00 ± 0.00	0.00 ± 0.00
*Sphingobacterium*	0.22 ± 0.19	0.00 ± 0.00	0.02 ± 0.02	0.08 ± 0.07
*Geodermatophilus*	0.26 ± 0.14	0.15 ± 0.11	1.30 ± 0.31	0.66 ± 0.02
*Alcaligenes*	0.25 ± 0.14	0.19 ± 0.07	0.10 ± 0.03	0.02 ± 0.01
*Oxalicibacterium*	0.19 ± 0.19	0.00 ± 0.00	0.04 ± 0.01	0.03 ± 0.01
*Candidatus Amoebophilus*	0.19 ± 0.18	0.02 ± 0.01	0.05 ± 0.03	0.25 ± 0.04
*Sphingomonas*	0.18 ± 0.15	0.01 ± 0.00	0.15 ± 0.07	0.11 ± 0.03
*Bradyrhizobium*	0.20 ± 0.12	0.09 ± 0.06	0.62 ± 0.47	0.84 ± 0.43
*Acinetobacter*	0.19 ± 0.12	0.22 ± 0.05	0.10 ± 0.05	0.01 ± 0.00
unclassified (derived from *Nitrosomonadales*)	0.16 ± 0.14	0.01 ± 0.00	0.12 ± 0.07	0.11 ± 0.00
*Nocardioides*	0.23 ± 0.07	0.18 ± 0.13	0.91 ± 0.11	0.20 ± 0.04
unclassified (derived from *Enterobacteriaceae*)	0.16 ± 0.14	0.19 ± 0.07	0.16 ± 0.13	0.20 ± 0.02
*Cystobacter*	0.17 ± 0.12	0.20 ± 0.18	0.06 ± 0.01	0.20 ± 0.04
*Cellulomonas*	0.16 ± 0.09	0.15 ± 0.15	0.17 ± 0.06	0.03 ± 0.01

Legend: *SHR* Sheila rhizosphere soil, *SHB* Sheila bulk soil, *ITR* Itsoseng rhizosphere soil, *ITB* Itsoseng bulk soil, data represent mean ± standard error of the relative abundance of microbial genera

### Influence of environmental factors on microbial communities

**Fig. 5:**
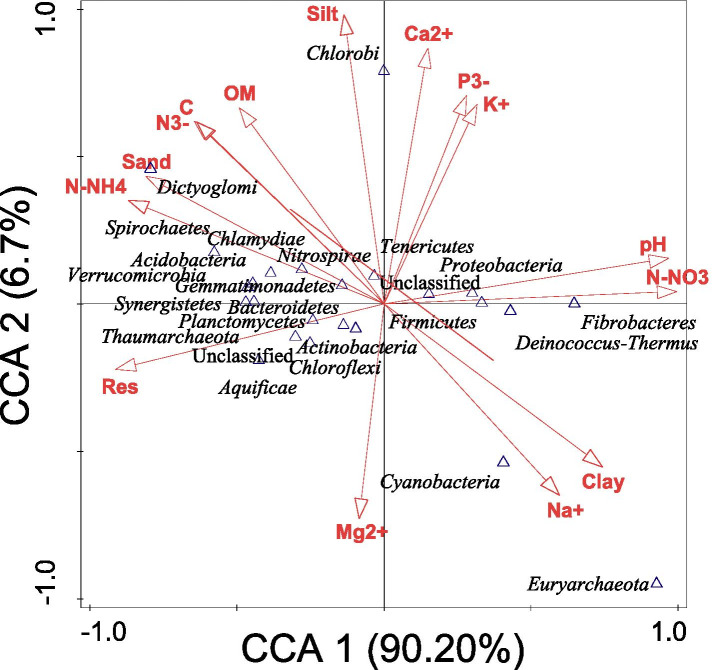
The influence of environmental variables on the microbial phyla from sunflower rhizosphere and bulk soils obtained from Sheila and Itsoseng using Canonical correspondence analysis

Canonical correspondence analysis (CCA) was used to determine the influence of the environmental variables on the relative abundance of microbial species at the phylum level. *Tenericutes* and *Proteobacteria* correlated positively with Ca^2+^, P^3−^, K^+^, pH, N-NO_3_ but negatively correlated with silt, OM, C, N^3−^, sand, N-NH_4_, Res, Mg^2+^, Na^+^, and clay. *Dictyoglomi*, *Chlorobi*, *Spirochaetes*, *Chlamydiae*, *Nitrospirae*, *Actinobacteria*, *Gemmatimonadetes*, and *Verrucomicrobia* positively correlated with N-NH_4_, sand, N^3−^, C, OM and silt and negatively correlated with Ca^2+^, P^3−^, K^+^, pH, N-NO_3_, clay, Na^+^, Mg^2+^ and Res. *Thaumarchaeota*, *Bacteroidetes*, *Planctomycetes*, *Actinobacteria*, *Aquificae* and *Chloroflexi* positively correlated with Res and Mg, but negatively correlated with N-NH_4_, sand, N^3−^, C, OM, silt, Ca^2+^, P^3−^, K^+^, pH, N-NO_3_, clay and Na^+^, while *Firmicutes*, *Cyanobacteria*, *Deinococcus*-*Thermus* and *Fibrobacteria* positively correlated with Na^+^ and clay but negatively correlated with N-NO3, pH, K^+^, P^3−^, Ca^2+^, silt, OM, C, N^3−^, sand, N-NH_4_, Res and Mg^2+^ (Fig. 5).

From the above analysis it was also observed that *Tenericutes* and *Proteobacteria* were positively influenced by SHB, *Dictyoglomi*, *Chlorobi*, *Spirochaetes*, *Chlamydiae*, *Nitrospirae*, *Actinobacteria*, *Gemmatimonadetes*, and *Verrucomicrobia* were positively influenced by ITB, *Thaumarchaeota*, *Bacteroidetes*, *Planctomycetes*, *Actinobacteria*, *Aquificae* and *Chloroflexi* were positively influenced by ITR, while *Firmicutes*, *Cyanobacteria*, *Deinococcus*-*Thermus* and *Fibrobacteria* were positively influenced by SHR. This further shows that SHB correlated positively with Ca^2+^, P^3−^, K^+^, pH, N-NO_3_ but negatively correlated with silt, OM, C, N^3−^, sand, N-NH_4_, Res, Mg^2+^, Na^+^, and clay. ITB positively correlated with N-NH_4_, sand, N^3−^, C, OM and silt and negatively correlated with Ca^2+^, P^3−^, K^+^, pH, N-NO_3_, clay, Na^+^, Mg^2+^ and Res. ITR positively correlated with Res and Mg^2+^, but negatively correlated with N-NH_4_, sand, N^3−^, C, OM, silt, Ca^2+^, P^3−^, K^+^, pH, N-NO_3_, clay and Na^+^, while SHR positively correlated with Na^+^ and clay but negatively correlated with N-NO_3_, pH, K^+^, P^3−^, Ca^2+^, silt, OM, C, N^3−^, sand, N-NH_4_, Res and Mg^2+^.

The environmental variables that best described or explained the difference observed in the microbial community compositions observed in Fig. 5 were determined using the forward selection and the Monte Carlo permutation test with random permutations. It was observed that out of the 15 variables considered, N-NO_3_ significantly (*p* < 0.1) contributed 94.6% of the difference, while N-NH_4_ significantly (*p* < 0.1) contributed 72.9% of the difference to the microbial community composition and abundance of the different soil samples (Table 5). On the contrary, although the remaining 13 variables contributed to the difference in the microbial phyla, their contributions were not significant (*p* ≥ 0.05).

**Table 5:** Forward selection of environmental variables that best described difference in microbial species between soil samples

Environmental variable	Explains %	Contribution %	Pseudo-F	*p* value
N-NO_3_ (mg/kg)	89.7	94.6	17.4	0.052
pH	85.0	90.8	11.3	0.18
Res (ohm)	75.8	82.9	6.3	0.276
N-NH_4_ (mg/kg)	69.3	72.9	4.5	0.048
Sand (%)	60.8	63.5	3.1	0.078
Clay (%)	52.1	53.3	2.2	0.18
Total N (%)	40.4	39.8	1.4	0.32
Total C (%)	39.7	39.0	1.3	0.33
Na^+^ (mg/kg)	35.3	29.9	1.1	0.454
OM (%)	25.7	23.1	0.7	0.646
K^+^ (mg/kg)	13.5	18.4	0.3	0.96
P^3−^ (mg/kg)	11.7	15.9	0.3	0.964
Ca^2+^ (mg/kg)	7.1	8.5	0.2	0.886
Silt (%)	7.1	3.4	0.2	0.668
Mg^2+^ (mg/kg)	5.5	6.0	0.1	0.966

### Soil functional measurements

**Fig. 6:**
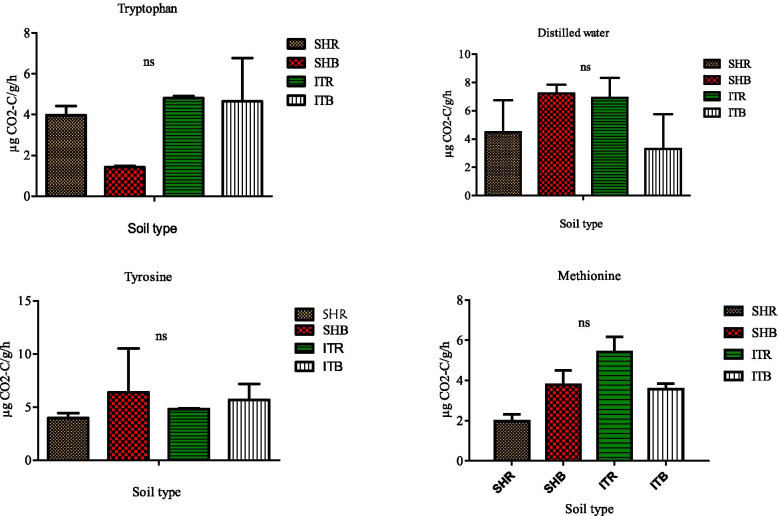
Effects of soil type on soil functional properties as measured for distilled water i.e., control and tryptophan, tyrosine and methionine using MicroResp assay. Number of replicates (n) = 2. Data represent mean ± SE, ns = not statistically significant following Duncan’s multiple range testleast significant difference test. SHR = Sheila rhizosphere soil, SHB=Sheila bulk soil, ITR = Itsoseng rhizosphere soil, ITB=Itsoseng bulk soil

**Fig. 7:**
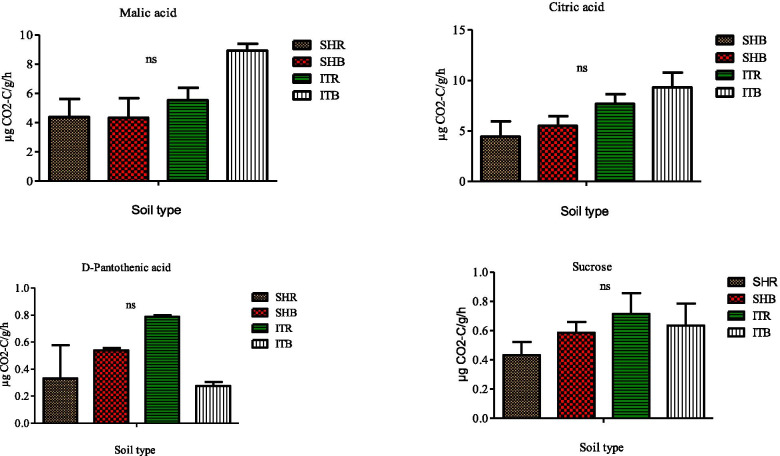
Effects of soil type on soil functional properties as measured for malic acid, D-pantothenic acid, citric, and sucrose using MicroResp assay. Number of replicates (n) = 2. Data represent mean ± SE, ns = not statistically significant following Duncan’s multiple range testleast significant difference test. SHR = Sheila rhizosphere soil, SHB=Sheila bulk soil, ITR = Itsoseng rhizosphere soil, ITB=Itsoseng bulk soil

**Fig. 8:**
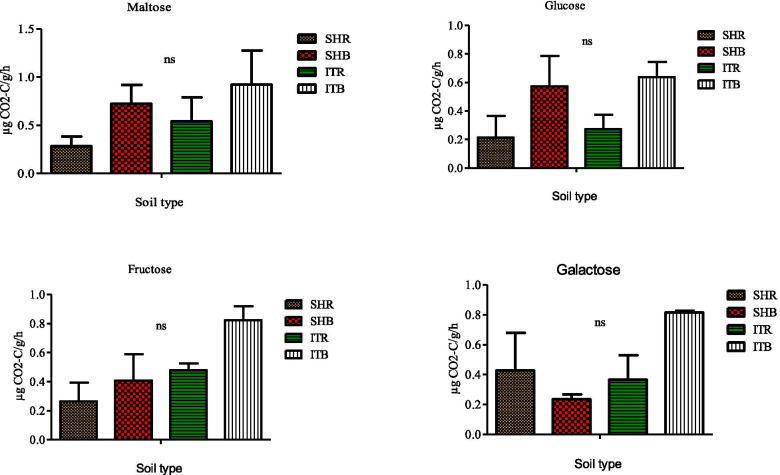
Effects of soil type on soil functional properties as measured for maltose, fructose, glucose, and galactose using MicroResp assay. Number of replicates (n) = 2. Data represent mean ± SE, ns = not statistically significant following Duncan’s multiple range testleast significant difference test. SHR = Sheila rhizosphere soil, SHB=Sheila bulk soil, ITR = Itsoseng rhizosphere soil, ITB=Itsoseng bulk soil

Individual substrate stimulated respiration, hence was determined by the respiration response of individual carbon compound which revealed the microbial communities present in the different soil types (SHR, SHB and ITR, ITB) showed different respiration rates (Figs. 6, 7 and 8). However, the microbial respiration rates for all the substrates used in this study were not statistically significant (*p* > 0.05). In particular, the respiration rate was higher under tryptophan amended soil for Sheila rhizosphere soil (2.33 ± 0.51 0 μg g^− 1^ h^− 1^ CO_2_-C) than Sheila bulk soil (1.42 ± 0.06 μg g^− 1^ h^− 1^ CO_2_-C), while Itsoseng rhizosphere soil (4.81 ± 0.100 μg g^− 1^ h^− 1^ CO_2_-C) was similarly higher than Itsoseng bulk soil (4.66 ± 2.13 μg g^− 1^ h^− 1^ CO_2_-C). On the contrary, Sheila (3.97 ± 0.44 μg g^− 1^ h^− 1^ CO_2_-C) and Itsoseng bulk (4.81 ± 0.10 μg g^− 1^ h^− 1^ CO_2_-C) soils were both higher than their respective rhizosphere soils when tyrosine was used as a substrate (Fig. 6). The respiration rate of microbial communities in Itsoseng rhizosphere soil was higher than Itsoseng bulk soil including Sheila rhizosphere and bulk soils under tyrosine amendment (Fig. 6).

When the amino acid, malic acid was applied as a substrate, the respiration rates of Sheila rhizosphere and bulk soils were similar since they both had related mean values of 4.38 ± 1.25 μg g^− 1^ h^− 1^ CO_2_-C and 4.35 ± 1.33 μg g^− 1^ h^− 1^ CO_2_-C respectively, while Itsoseng bulk soil with a mean value of 8.92 ± 0.48 μg g^− 1^ h^− 1^ CO_2_-C was higher than its rhizosphere soil with a mean value of 5.53 ± 0.84 μg g^− 1^ h^− 1^ CO_2_-C (Fig. 7). A similar result pattern was observed for both rhizosphere and bulk soils in Sheila and Itsoseng when citric acid was used as a carbon substrate. Contrarily, the resulting pattern observed in D-pantothenic acid and sucrose amended soil was almost similar, except that D-pantothenic acid amended bulk soil of Itsoseng was relatively lower than the other soil types (Fig. 7).

**Fig. 9:**
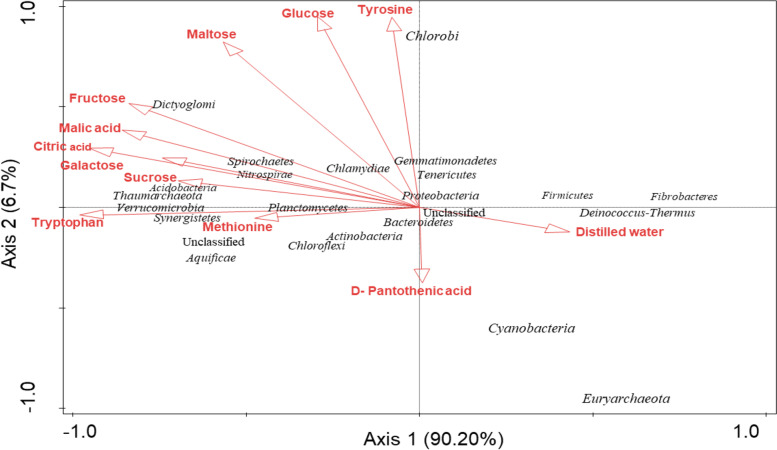
The influence of carbon substrates on the microbial phyla from sunflower rhizosphere and bulk soils obtained from Sheila and Itsoseng using Canonical correspondence analysis (CCA)

However, when the carbon substrates maltose, fructose, glucose and galactose were used, a different set of results was obtained (Fig. 8). In that it was only in the galactose amended rhizosphere soil (0.43 ± 0.25 μg g^− 1^ h^− 1^ CO_2_-C) of Sheila, that had a higher microbial respiration rate than its bulk soil (0.24 ± 0.03 μg g^− 1^ h^− 1^ CO_2_-C) (Fig. 8). CCA was used to determine the influence of the carbon substrates on the relative abundance of microbial species at the phylum level (Fig. 9). *Spirochaetes*, *Chlamydiae*, *Nitrospirae*, *Gemmatimonadetes, Acidobacteria, Thaumarchaeota, Dictyoglomi*, and *Viromicrobia* positively correlated with tyrosine, glucose, maltose, fructose, malic acid, citric acid, galactose and sucrose but negatively correlated with tryptophan, methionine, D-pantothenic acid, distilled water. *Synergistetes*, *Actinobacteria*, *Chloroflexi*, *Aquificae*, *Bacteriodetes* positively correlated with tryptophan, methionine and D-pantothenic acid but negatively correlated with tyrosine, glucose, maltose, fructose, malic acid, citric acid, galactose, sucrose and distilled water, while *Euryarchaeota*, *Cyanobacteria* and *Deinococcus*-*Thermus* positively correlated with distilled water but negatively correlated with tyrosine, glucose, maltose, fructose, malic acid, citric acid, galactose, sucrose, tryptophan, methionine and D-pantothenic acid. However, none of the carbon substrates influence the abundance of *Fibrobacteres, Firmicutes, Tenericutes* and *Proteobacteria* (Fig. 9).

## Discussion

In this present study, we have shown differences between two soil types from Sheila and Itsoseng by profiling the microbial diversity associated with sunflower rhizosphere soils and bulk soils using 16S rDNA amplicon sequencing technique. Differences in functional measurements between the two soil types from the different locations were determined using CLPP approach. Community respiration and substrate-induced respiration have been constantly used to assess ecosystem function because it is broadly distributed among diverse groups of microbes and are considered as a suitable alternative for total biological activity [28, 29].

Considering the microbial diversity in this study, rarefaction curves were plotted to depict the microbial richness of each sample. The two soil samples with the highest species counts were obtained from ITB (light green curve) and ITR (purple curve), even though soil sample from SHB (orange curve) had the highest number of reads (120,000). In agreement with [30], the analysis from the rarefaction curves obtained in this study had shown that the majority of the read numbers from all the soil samples reached saturation, indicating that the sampling method sufficiently captured a high proportion or percentage of the microbial species from the different sampling sites. In addition, reductions in bp count of the sequence read in all the soil samples were observed (Table 2). This reduction in bp count is as a result of the removal of low quality and contaminating sequences, which could affect downstream analysis [31].

It was further observed that the sampling sites vary in physical and chemical properties and alpha diversity. Variations in the microbial community composition among soil types using PCoA were also observed. This indicates that the microbial community structures are perhaps unique to the soil type. This was further buttressed statistically by the analysis of similarity (ANOSIM), which shows that the separation was significant (*p*-value = 0.01, R = 0.60). This significant variation can be attributed to the significant differences (*p* ≤ 0.05) recorded for most of the physical and chemical properties of the soil types, and this is similar to the result reported by [2]. The uniqueness of microbial species in the different soil types could be due to restricted dispersal mechanisms between the two sunflower farms as well as local assembly mechanisms that may remove or introduce certain microbial species within a particular soil type [32]. The alpha diversity of microbial taxonomic groups of (Simpson, Shannon, and evenness indicators) of the two sunflower farms soil types were not significantly different, and this is similar to the report of [30]. Previous studies on microbes associated with plants have demonstrated that soil type plays a major role in shaping microbial diversity structure [5, 33–35]. Findings from this study however differ from the findings of [36], who highlighted no difference or separation in beta diversity of soil types or samples when they considered the conserved regions of the microbial species through next generation sequencing approach.

At the phylum level, we observed that *Firmicutes* predominated in the soil type SHR with a mean relative abundant value of 39.74. Also, abundant at the phylum level included *Actinobacteria* with a mean relative abundant value of 26.41 in the soil type ITR (Table 3). The high abundance of *Firmicutes* and *Actinobacteria* can partly be explained by the influence of the sunflower root exudates within the sunflower rhizosphere region [1]. These two phyla were among the core microbiome reported by [6] in soybean plants’ rhizosphere inoculated with *Rhizobium* species and mycorrhizal fungal consortium. Most of the bacteria in these two groups possess the particular traits of promoting plant growth because they are endowed with plant growth-promoting traits [4, 6]. These plant growth-promoting traits may include, but not limited to, siderophores and exopolysaccharide production, phosphate solubilization, hydrogen cyanide (HCN) production, and indole-acetic-acid (IAA) production [4, 37, 38]. In a study conducted by [39] using Illumina sequencing approach, *Actinobacteria*, *Acidobacteria*, *Verrucomicrobia*, *Planctomycetes*, *Proteobacteria*, and *Bacteroidetes* were present in both rhizosphere and bulk soils of sunflower plants. This is, however, in contrast with the work of [2] who reported *Proteobacteria* as the dominant keystone microbe in the rhizosphere of sunflower plants.

Microbes with plant growth-promoting functions were also revealed at the genus level. In particular, SHR and ITR predominantly harbored more *Bacillus* and *Arthrobacter,* respectively than their corresponding SHB and ITB (Table 4). It was, however, observed that the genus *Candidatus Nitrososphaera* was low in SHR and SHB compared to ITR and ITB (Table 4). This result is parly similar to the work of [2] who reported a significant reduction of *Nitrososphaera* in the rhizosphere of sunflower compared to the bulk soil. *Nitrososphaera* is known to be involved in nitrification process [40]. Nitrification may either be induced or inhibited in the rhizosphere [1], which can eventually affect nitrate and ammonium availability in the rhizospheres. Also, the genera *Aspergillus*, *Alternaria*, *Penicillium*, and *Fusarium* were the dominant fungi found in the rhizosphere of sunflower plants amended with organic manure [41]. Interestingly, in this study, we discovered a relatively high proportion of unclassified microbes at the phylum, and genus levels, even after using other bioinformatics and clustering tools besides MG-RAST such as Quantitative Insights into Microbial Ecology (QIIME) and mothur. It is possible that these unclassified microbes would contain useful plant-microbial functions. Thus, isolating and cataloging these microbes may lead to the discovery of new species with novel plant growth functions. This will help to bridge the existing lacuna in food production, as such new species could be used to complement and/or replace conventional chemical fertilizer application for sustainable agriculture.

The CCA results showed that the phylum *Firmicutes*, as well as other phyla like *Cyanobacteria*, *Deinococcus*-*Thermus* and *Fibrobacteres,* positively correlated with Na^+^ and clay but negatively correlated with N-NO_3_, pH, K^+^, P^3−^, Ca^2+^, silt, OM, C, N^3−^, sand, N-NH_4_, Res and Mg^2+^. This means that an increase in the amount of Na^+^ and clay in the soil would directly translate to an increase in the abundance of these microbes, while an increase in the amount of N-NO_3_, pH, K^+^, P^3−^, Ca^2+^, silt, OM, C, N^3−^, sand, N-NH_4_, Res and Mg^2+^ would lead to a decrease in the abundance of the microbes. Therefore, the relatively high amounts of Na^+^ (78.70%) and clay (20%) that were present in SHR (Table 1) could have contributed to the dominance of *Firmicutes* in SHR. It may appear that these two environmental variables (Na^+^ and clay) did not have an influence on *Cyanobacteria*, *Deinococcus*-*Thermus* and *Fibrobacteres* because their mean relative abundance values were relatively low in this study. The effects of Na^+^ and clay on the latter microbes may not be obvious due to other external factors that might have played crucial roles in determining their occurrence and/or abundance, as was previously reported by [42]. Disentangling the impact of soil type on microbial composition is still a challenge under field conditions. The soil’s physical and chemical properties affect the soil microbiota as well as agricultural management practices, weather conditions (including relative humidity, temperature, and amount of rainfall) [6], and cropping history. This study also showed that the occurrence and/or abundance of *Actinobacteria*, *Thaumarchaeota*, *Bacteroidetes*, *Planctomycetes*, *Aquificae* and *Chloroflexi* were positively influenced by Res and Mg^2+^, and negatively affected by N-NH_4_, sand, N^3−^, C, OM, silt, Ca^2+^, P^3−^, K^+^, pH, N-NO_3_, clay and Na^+^. A similar result was observed in another study where Mg^2+^ influenced phylogenetic diversity of sunflower rhizosphere but not in sorghum rhizosphere soils [2]. However, this study is different from the study of [2] given that, in this present study, Ca^2+^, P^3−^, and K^+^ negatively impacted the microbial community composition of the sunflower rhizosphere, whereas Ca^2+^, P^3−^ and K^+^ positively contributed to the sunflower rhizosphere microbial community reported by [2].

However, from the CCA results, it was found that N-NO_3_, pH, Res, sand, clay, total N^3−^, total C, Na^+^, OM, K^+^, P^3−^, Ca^2+^, silt and Mg^2+^ did not significantly (*p >* 0.05) enhance soil microbial composition, while N-NO_3_ and N-NH_4_ were the only environmental variables that significantly improved microbial community structure and composition since they respectively showed 94.6 and 72.9% significant contribution (*p* < 0.1) to microbial species (Table 5), particularly in ITB. These findings contradict the findings of [30], who reported significant contributions of 46% (from pH) and 11.50% (from sand) to soil bacterial communities. Nevertheless, all the soil physical and chemical properties considered played some roles in shaping the microbial communities.

Considering the functional diversity of the microbial communities in sunflower rhizosphere and bulk soils, the soil microbial communities utilized the various carbon substrates at different rates. The consumption rate of these substrates is used for characterizing microbial species [43]. Using the relative abundance values at the phyla level and the measured respiration rate or functional property values, the influence of carbon source on the community composition was done using the statistical software canonical correspondence analysis. From the analysis, the data show the ability of some microbes to metabolize the incorporated carbon substrates. The analysis unveiled that the microbial communities in SHR and ITR used the amino acid tryptophan more efficiently, but malic acid was more effectively utilized by microbial species in SHR. Similarly, carbohydrate substrates were observed to be proficiently used by the microbial communities, particularly those of SHR, which metabolized galactose more effectively. The CCA analysis further revealed that the effectiveness in the metabolism of tryptophan by the microbial communities could have alluded to the relatively high abundance of *Actinobacteria* that was present in ITR.

Besides *Actinobacteria*, other phyla that positively contributed to the metabolism of tryptophan included *Synergistetes*, *Chloroflexi*, *Aquificae*, and *Bacteriodetes.* The CCA analysis however showed that these microbial phyla did not positively contribute to the metabolism of tyrosine, glucose, maltose, fructose, malic acid, citric acid, galactose, and sucrose. The analysis also showed that *Spirochaetes*, *Chlamydiae*, *Nitrospirae*, *Gemmatimonadetes, Acidobacteria, Thaumarchaeota, Dictyoglomi*, and *Viromicrobia* positively correlated with tyrosine, glucose, maltose, fructose, malic acid, citric acid, galactose and sucrose but negatively correlated with tryptophan, methionine, D-pantothenic acid and distilled water. These positive correlations may partly be due to the usage of the carbon substrates as an energy source by soil microbes [44]. Some of these carbon substrates, such as carbohydrates, are the abundant organic compounds in the soil. It has been postulated that the most critical roles of sugars in the soil are the preservation and stimulation of microbial activities for priming results [44, 45]. Amino acid, which is majorly used for building up microbial biomass, was the least respired among some of the substrates used. The low amounts of nitrogen in the soils give an explanation for this. Consequently, it can be considered that a major percentage of the amino acids available in the soils were assimilated for building up microbial biomass, and only a small quantity was used for respiration since there was a low level of N content available in the soils [24, 45].

## Conclusion

Using 16S rDNA amplicon sequencing approach, this study has shown that *Firmicutes*, *Actnobacteria* and *Proteobacteria* predominated sunflower rhizosphere soils. *Firmicutes*, as well as other phyla like *Cyanobacteria*, *Deinococcus*-*Thermus* and *Fibrobacteres* were positively influenced by Na^2+^ and clay, while *Actinobacteria*, *Thaumarchaeota*, *Bacteroidetes*, *Planctomycetes*, *Aquificae* and *Chloroflexi* were positively influenced by Res and Mg^2+^. We also report that N-NO_3_ and N-NH_4_ significantly contributed to the microbial communities resulting in the differences observed in the sunflower rhizosphere and bulk soils. The CLPP analysis further demonstrated that microbial communities in SHR and ITR used the amino acids tryptophan and malic acid more efficiently and that the relatively high metabolisms of these carbon substrates may be due to the dominant nature of some of the microbes (e.g., *Actinobacteria*) in the soils. It was also observed that the number of identified rRNA features was larger than the number of sequences, and this could be linked to the high proportion of unclassified bacteria observed in this study since it has been reported that application of rRNA genetic information helps to identify novel sequences, predict the nutritional composition for bacteria that are un-culturable and improve media formulation or development [46, 47]. With the high proportion of unclassified microbes observed in the studied soils, we therefore recommend that efforts should further be made to isolate, characterize and identify these unclassified microbial species, as it might be plausible to discover new microbial candidates that can further be harnessed for biotechnological purpose.

## Data Availability

The data are available in NCBI database under Bioproject number PRJNA672856 for samples, SH samples- SHR (S1_A and S2_B) and SHB (S3_C and S4_D) and IT samples, ITR (S5_M and S6_N) and ITB (S7_O and S8_P). The samples can be accessed under SRA accession numbers SRR12960272, SRR12960271, SRR12960268, SRR12960267, SRR12960266, SRR12960265, SRR12960264 and SRR12960263, respectively. The quality-filtered and annotated data for individual replicates have been released publicly in the MG-RAST database with the accession numbers mgs831279 (sample S1_A), mgs831282 (sample S2_B), mgs831285 (sample S3_C), mgs831300 (sample S4_D), mgs831303 (sample S5_M), mgs831306 (sample S6_N), mgs831309 (sample S7_O) and mgs831312 (sample S8_P).

## Abbreviations

ANOVA: Analysis of Variance
BLAST: Basic Local Alignment Search Tool
CCA: Canonical correspondence analysis
CLPP: Community level physiological profiling
DNA: Deoxyribonucleic acid
NCBI: National Centre for Biotechnology Information
OD: Optical density
HCN: Hydrogen cyanide
IAA: Indole-acetic-acid
ITB: Itsoseng soil
ITR: Itsoseng rhizosphere soil
N-NH_4_: Ammonium
N-NO_3_: Nitrate
NS: Not statistically significant
PAST: Paleontological statistics software package
PCoA: Principal coordinates analysis
PCR: polymerase chain reaction
PerMANOVA: Permutational multivariate analysis of variance
QIIME: Quantitative Insights into Microbial Ecology
RAST: Rapid annotation using subsystem technology
RES: Resistivity
SE: Standard error
SHB: Sheila bulk soil
SHR: Sheila rhizosphere soil
SRA: Sequence Read Archive
